# A case of extranasopharyngeal angiofibroma arising from nasal dorsum

**DOI:** 10.1016/j.bjorl.2024.101508

**Published:** 2024-09-11

**Authors:** Tae-Gyun Kim, Chang-Ho Whangbo, Mi Kyung Ye, Seung-Heon Shin

**Affiliations:** Daegu Catholic University, School of Medicine, Department of Otorhinolaryngology — Head and Neck Surgery, Daegu, Republic of Korea

## Introduction

Angiofibroma is a benign but potentially locally aggressive vascular tumor that typically originates in the nasopharynx. The etiology of Juvenile Nasopharyngeal Angiofibroma (JNA) is not completely understood.^1^ These tumors commonly arise from the superior aspect of the sphenopalatine foramen at the posterolateral wall of the nasal cavity and typically develop in adolescent males. Histologically, they are characterized by non-encapsulated fibrous connective stromal tissue with abundant vascular tissue and sporadically contain smooth muscle cells in their wall. Due to its aggressive nature, complete surgical resection is important for a good prognosis and to minimize significant morbidity.

Extranasopharyngeal Angiofibroma (ENA) is an atypical form of angiofibroma with same histologic appearance, but different clinical characteristics compared to JNA. While JNA commonly occurs in adolescent males, ENA can affect both sexes and can develop in very young as well as elderly patients.^2^ The symptoms of ENA vary depending on the site of origin. Complete surgical removal is the therapy of choice for ENA. Although the results of surgery and surgical approach for JNA vary depending on the size, location, tumor stage, and blood supply of the tumor, most of ENA can be treated with minimal postoperative morbidity and recurrence.^1^, ^2^ Most of ENA cases arise from the nasal cavity, with nasal septum being the most common site, followed by the maxillary and ethmoid sinuses.^2^, ^3^ ENA located in the nasal dorsum is extremely rare, and in this report, we present a case of ENA located at the nasal dorsum supratip lobule area.

## Case report

A 12-year-old male visited our department with a history bulging in the left supratip lobule area for 4 months. He did not have any discomfort and history of trauma, bleeding tendency, or infection. Nasal endoscopy revealed no abnormal findings in the nasopharynx, nasal septum, or turbinates.

**Figure 1:**
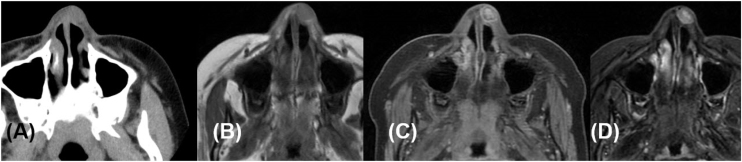
CT scan (A), T1 (B), contrast-enhanced T1 (C), and T2 (D) MRI show well demarcated ovoid benign mass lesion, measuring 14 × 12 mm in size at the left nasal dorsum with inhomogeneous hyperintensity in T2 image.

Computed Tomography (CT) and Magnetic Resonance Imaging (MRI) of the paranasal sinuses revealed a well-defined, T2-hyperintense benign mass lesion measuring 12 mm in the left supratip area, with marginal enhancement on contrast-enhanced T1 images. There were no abnormal findings in sinuses and nasal cavity (Fig. 1).

**Figure 2:**
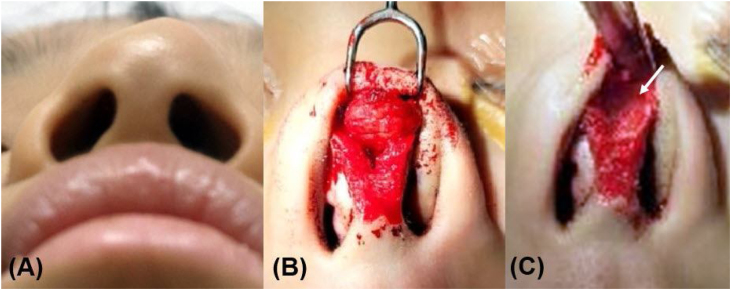
Clinical and surgical photos. (A) The left nasal dorsum area was bulged. (B) A round mass was located above the junctional area of the left lateral crus of the alar and upper lateral cartilage. (C) Depressed left lateral crus (arrow) of the alar was observed after mass removal.

**Figure 3:**
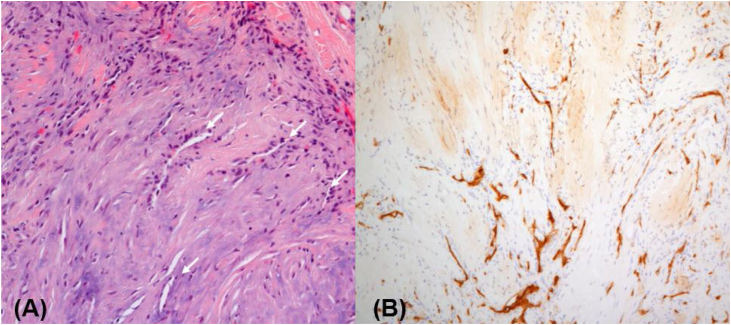
Histopathological findings. (A) Hematoxylin eosin stain showed multiple non-dilated vascular structures (arrows) with absent muscular layer and dense fibro-collagenous lesions without atypical cells (×200). (B) Immunohistochemical stain for CD34 demonstrated marked hypervascularity in the dense fibrous stroma (×100).

**Figure 4:**
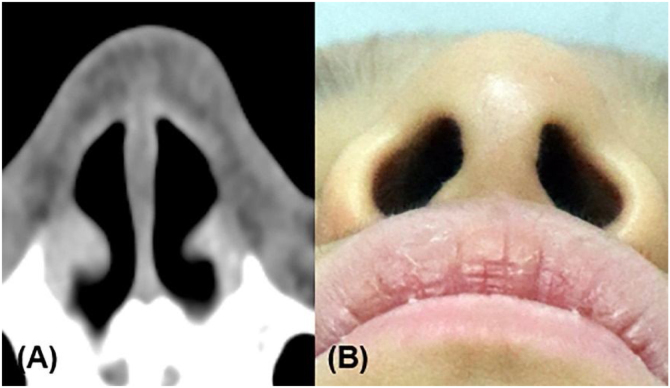
Post-operative 1-year CT scan (A) and clinical photo (B) revealed no evidence of recurrence or residual lesion.

The mass was removed en bloc from between the perichondrium and the subcutaneous musculoaponeurotic system using an open rhinoplasty approach. A well-encapsulated, reddish, rubbery mass measuring 14 × 12 mm was located above the junctional area of the left lateral crus of the alar and upper lateral cartilage (Fig. 2). No significant bleeding was observed during surgery. Histologic examination of the cut surface showed focal hemorrhagic foci. Hematoxylin and eosin staining revealed dilated cavernous vascular space with fibrosis, positive smooth muscle actin staining, and approximately 5% of cells staining Ki-67 positive. These findings were compatible with a diagnosis of angiofibroma (Fig. 3). A follow-up CT scan 12 months after surgical excision showed no signs of recurrence, with intact alar and upper lateral cartilage (Fig. 4).

## Discussion

ENA are rare vascular tumors with clinical and radiological characteristics distinct from those of JNA. ENA can develop without sexual predominance and can be observed in individuals of all age.^2^ In 2017, Windfuhr and Vent reviewed 174 ENA patients, finding that 118 patients presented with lesions in the nasal and sinonasal spaces, and only one case was located at nasal dorsum.^2^, ^4^ However, in that case, the mass arose from the junction between the septum and alar cartilage and continued as a swelling in the nasal dorsum. Although the authors suggested that the ENA was localized in the nasal dorsum, it might have originated from the nasal septum. We believe this case is the first reported instance of ENA developed in the nasal dorsum supratip lobule.

The clinical symptoms of ENA patients depend on location of the tumor. ENAs developing in the nasal or sinus cavity of present with nasal obstruction and epistaxis, and sometimes without any symptoms. Compared to JNA, ENA tends to grow faster, with symptoms developing within 12 months or less before diagnosis.^2^, ^5^ In this case, the ENA developed between the perichondrium and the subcutaneous musculoaponeurotic system, with the patients experiencing no discomfort other than the visible mass in the nasal dorsum area.

Before surgical intervention for JNA, CT scan and MRI are essential for evaluating the extension and vascularity of the tumor. The vascularity of ENA varies from strong to minimal or even no enhancement. In this patient, CT scan and MRI finding showed a non-enhancement and well-demarcated benign mass. Radiologist even suggested it could be a chondroma. Although the feeding vessel of the tumor could not be identified, considering location of the tumor, it is likely to be the external nasal branch of the anterior ethmoidal artery. The nasal dorsum is a very rare location for angiofibroma, and the patient did not present with typical symptoms such as nasal obstruction, with or without nasal bleeding. Therefore, it is necessary to differentiate this condition from other disease entities, such as inflammatory or angiomatous polyps, cysts, chondroma, and benign or malignant tumors.

Complete surgical excision is the treatment of choice for both JNA and ENA. In case of JNA, the surgical approach is determined by the size and location of the tumor. If complete removal cannot be achieved through surgery, hormonal therapy, radiation therapy, and chemotherapy can be applied. However, most of the ENA cases can be completely removed with an endoscopic or external approach. Among the 174 cases of ENA, about 43.7% were treated with a transnasal approach and only on case was treated with open rhinoplasty approach.^2^ In this case, we completely removed the tumor using the open rhinoplasty approach. Approximately 25% of JNA patients require revision or additional therapy with tumor recurrence. In contrast to JNA, recurrence rate of ENA was about 2.3% within 12 months after treatment.^2^ Our case did not show any evidence of recurrence with CT scan after 12 months.

## Conclusion

ENA is a rare vascular tumor originating from various lesions in the head neck area. Histologically it is very similar to JNA, but its clinical, radiologic, and demographic characteristics are different. ENA can develop at any age, in both sexes, and can arise in various regions of the head and neck. This is the first reported case of ENA found on the nasal dorsum, successfully treated through an open rhinoplasty approach. Although ENA is uncommon, it is important to consider that semisolid lesions in the nasal and sinonasal area could be ENA during differential diagnosis.

## Funding

The author(s) received no financial support for the research, authorship, and/or publication of this article.

## Conflicts of interest

The authors declare no conflicts of interest.
